# Pandemic elevates sensitivity to moral disgust but not pathogen disgust

**DOI:** 10.1038/s41598-023-35375-2

**Published:** 2023-05-22

**Authors:** Dagmar Schwambergová, Šárka Kaňková, Jitka Třebická Fialová, Jana Hlaváčová, Jan Havlíček

**Affiliations:** 1 Department of Zoology, Faculty of Science, Charles University, Viničná 7, 128 44 Prague 2, Czech Republic; 2 National Institute of Mental Health, Topolová 748, 250 67 Klecany, Czech Republic; 3 Department of Philosophy and History of Science, Faculty of Science, Charles University, Viničná 7, 128 44 Prague 2, Czech Republic

**Keywords:** Psychology, Human behaviour

## Abstract

The behavioral immune system, with disgust as its motivational part, serves as the first line of defense in organisms’ protection against pathogens. Laboratory studies indicate that disgust sensitivity adaptively adjusts to simulated environmental threat, but whether disgust levels similarly change in response to real-life threats, such as a pandemic, remains largely unknown. In a preregistered within-subject study, we tested whether the threat posed by the Covid-19 pandemic would lead to increased perceived disgust. The perception of threat was induced by testing during two phases of the Covid-19 pandemic (periods of high vs. low pathogen threat). We found heightened levels of moral disgust during a “wave” of the pandemic, but the effect was not observed in the domain of pathogen or sexual disgust. Moreover, the age of respondents and levels of trait anxiety were positively associated with pathogen and moral disgust, suggesting that variation in disgust sensitivity may be based chiefly on stable characteristics.

## Introduction

Immunological defense plays a crucial role in our resistance against pathogens, but the physiological immune system is also energetically costly and metabolically demanding. These demands can be reduced by activation of a behavioral immune system (BIS), which consists of psychological mechanisms directed at pathogen detection. They activate an affective and cognitive response which may lead to pathogen avoidance and hygienic behavior aimed at minimizing contagion risks^1^. The motivational (affective) component of the BIS is disgust^2^, frequently accompanied by physiological responses, such as nausea or lowered blood pressure^3^. Tybur and colleagues^4^ proposed that disgust consists of three functionally specific domains: moral (motivates avoidance of violators of social norms), sexual (motivates avoidance of sexual partners and behaviors which would jeopardize one’s long-term reproductive success), and pathogen disgust. The last-named is the most crucial domain for avoidance of possible contagion. While pathogen avoidance is a cross-cultural phenomenon and detection of cues to sickness is not restricted to in-group individuals^5^, there is a high interindividual and intraindividual variation in disgust sensitivity, influenced by numerous factors including gender^6^, age^6,7^, reproductive status^8^, and immunological vulnerability^9^.

Disgust sensitivity varies also depending on external factors, such as environmental risks. For example, a recent study by Cepon-Robins et al.^10^ showed that in Ecuadorian Shuar communities, which live in an environment with a high pathogen risk, higher pathogen disgust sensitivity led to lower infection levels. But even in generally low pathogen risk environments, epidemics do occur, and the BIS is expected to react flexibly to protect the organism. The Covid-19 pandemic, which currently poses a serious global threat^11^, represents such a situation, and emerging evidence is showing activation of the BIS via elevated disgust sensitivity. For example, Milkowska et al.^12^ compared disgust sensitivity in Polish women before and during the pandemic: during the pandemic, respondents reported higher disgust sensitivity to disgust-evoking pictures, but such increase was not observed in the pathogen domain of the Three-Domain Disgust Scale (TDDS). In another study, the germ aversion subscale of the Perceived Vulnerability to Disease Questionnaire (PVD) and the pathogen domain of TDDS were strongly associated with Covid-19 concerns and preventive behaviors such as social distancing, mask wearing, and cleaning^13^. Moreover, Australian students who completed a questionnaire during a lockdown reported higher disgust sensitivity than students who completed the same questionnaire before the pandemic^14^. All existing research, however, compares the scores of different groups of participants and comparability of the groups might be an issue. There is, therefore, a need to study this issue using a within-subject design that would monitor intraindividual fluctuations depending on the level of pathogen threat.

Gender and age seem to be the most prominent variables influencing disgust sensitivity. While the results for gender are unequivocal, with women showing on average higher disgust sensitivity than men do^6,15^, the effect of age is far less clear. For instance, Curtis et al.^6^ showed images with a potential disease threat to participants and found that the level of disgust was decreasing with age. Similarly, older participants scored lower on disgust sensitivity^7^. Other studies, however, report a positive association between both age and food disgust^16^ and age and germ aversion, although perceived infectability decreased up to the age of fifty^15^.

This study’s aim was to test whether the threat caused by the Covid-19 pandemic leads to increased perceived disgust as a major variable linked to the BIS. We employed a mixed research strategy combining an experimental design (a priming vignette, which is a common tool used in research on sexual and moral disgust^17–19^) and a naturally occurring event (the Covid-19 pandemic). The perception of threat was experimentally induced by a priming story (between-subject design). Each participant was tested twice (within-subject design): once during the first lockdown (high pathogen threat) and once over a year after the onset of pandemic, at a time when restrictions were lifted (low pathogen threat). Based on data from the National Institute of Public Health, we know that in the Czech Republic, the 21-day cumulative number of reported Covid-19 cases per 100,000 persons was 44 during the period of high pathogen threat and a strict lockdown (with 115–377 new cases a day in March–April 2020) and 25 during the period of low pathogen threat after most restrictions were lifted (with 41–208 new cases a day in June–July 2021). In line with preregistration, we expected higher scores in pathogen and moral disgust during the high pathogen threat period irrespectively of the priming. We also predicted that experimental priming would elevate the scores of disgust measures during the period of low, but not high pathogen threat due to a predicted ceiling effect. We expected no changes in trait anxiety, or general health status between the two periods but predicted that scores of trait anxiety and health-related questionnaire scores would positively modulate changes in disgust. Based on data from our pilot study, we also expected age to positively correlate with the pathogen and moral disgust domains during the period of high pathogen threat but not during the low pathogen threat.

## Materials and methods

The project was conducted online as a prospective study in the Czech Republic and included two data collection points at which participants were exposed to the same (experimental or control) priming conditions and completed the same set of questionnaires. The study was approved by the Institutional Review Board of Charles University (approval number 2020/02) and had been performed in accordance with the Declaration of Helsinki. Informed consent was obtained from all participants. Our predictions and the design were preregistered prior to accessing the data (https://osf.io/tvmxg).

### Power analysis

Statistical power analysis was computed in G*Power (Version 3.1)^20^. It was a priori specified that we would need a sample size of 180 participants to detect a medium effect size (d = 0.53; computed as a mean of effect sizes from relevant studies^21–23^) with an alpha of 0.05 at both data collection points. Based on our previous studies, we estimated that around 50% of respondents from the first round would be willing to participate in the second one (i.e., provide their email address). We further expected that around 75% of those who provide the email address would complete the study. Our target was thus to recruit at least 700 participants in the first round to have around 260 participants in the second round.

### Participants

We recruited participants (aged over 18 years) using a snowball sampling method. Respondents were contacted online, via Facebook sites and emails, and invited to participate in a study called Personality and Disgust. A total of 1047 respondents took part in the first round but 286 individuals were excluded from further analyses, mostly because they failed to complete the questionnaire (over one-fifth of the questionnaire unanswered). This resulted in a final sample size of 761 respondents (586 women and 175 men; women: M_age_ = 33.1, SD = 11.8; men: M_age_ = 33.4, SD = 10.6) in the first round, i.e. during the first “wave” of the Covid-19 pandemic. Fourteen months later, we recontacted participants who expressed interest in further participation and provided their email addresses (N = 442). In the second round, 249 participants took part but 25 were excluded because they did not complete the questionnaire. This resulted in a final sample size of 224 participants (175 women and 49 men; women: M_age_ = 33.0, SD = 10.9; men: M_age_ = 33.4, SD = 12.4). Participation in the study was conditional on informed consent and no financial reward was offered to respondents.

### Procedure

The first round of the study was conducted at the beginning of the Covid-19 pandemic. Data collection lasted three weeks, from March 22 to April 11, 2020, during the first lockdown in the Czech Republic. The second round was conducted after most restrictions had been (temporarily) lifted, between June 11 and July 4, 2021, when the pathogen threat substantially decreased. The priming consisted of two stories: (i) a story about people who became infected with Covid-19 and violated the quarantine (this focused on the threat of contagion and moral disgust and served as the experimental condition), and (ii) a story about the meltdown of a famous iceberg in Iceland (this concentrated on the threat posed by global warming and served as the control condition). To obtain balanced sample sizes for each priming condition, all even-numbered participants were in this first round assigned to experimental priming and all odd-numbered participants to the control priming condition. During the second round of data collection, all respondents were exposed to the same priming condition as they were in the first round.

At the beginning, respondents completed a sociodemographic inventory, inventory on long-term and current health, Personality Inventory, and Trait Anxiety Inventory. Responses provided for the initial set of inventories were not expected to fluctuate because they aimed at assessment of traits and their order was therefore not randomized. After the initial part, we displayed the priming story. Priming was followed by a set of questionnaires in a randomized order (State Anxiety Inventory, Perceived Stress Scale, Three Domain Disgust Scale, Culpepper Disgust Image Set, Body Odor Disgust Scale, and Hygienic Behavior). Participants also completed the Social Phobia Inventory and Xenophobia Inventory. At the end of the survey, participants completed a priming-related memory test. The results of the State Anxiety Inventory, Perceived Stress Scale, Personality Inventory, Hygienic Behavior Inventory, Social Phobia Inventory and Xenophobia Inventory will be presented elsewhere.

Moreover, we monitored the epidemiologic situation in the Czech Republic and recorded it in a “Covid diary,” which was updated daily based on information published on the website of the Ministry of Health and novinky.cz (a large online news provider in the Czech Republic). We recorded the number of new reported Covid-19 cases and deaths, as well as changes in local epidemiological restrictions. We started keeping the diary one month before launching the first round and ended one month after finishing the second round of the study. The “Covid diary” served as a control for the epidemiological situation mainly during data collection: we wanted to keep track of any significant changes in the epidemiological situation.

### Priming stories

The priming story in the experimental condition aimed at evoking the threat of contagion (pathogen disgust) and violation of rules (moral disgust). The main characters, a married couple, go skiing in northern Italy at the beginning of the coronavirus pandemic and become sick with Covid-19 a few days after their return. Although the husband is hospitalized, his wife violates the quarantine and visits her neighbors, an elderly couple. Later, one of the neighbors falls ill, develops severe pneumonia, and has to be put on artificial ventilation. The story was based on a common route of coronavirus spread from returning travelers to the Czech Republic and it took into account the increased risks Covid-19 poses to older persons. For details, see the P1 in the Supplementary materials.

In the control priming story, a married couple travel to Faxaflói, Iceland, to cross the famous iceberg Okjökull only to discover that there is no snow and temperatures are relatively high. They wait for a few days, but the situation does not improve, so they decide to return home earlier. The wife visits her neighbors, an elderly couple, who tells her that the Okjökull is melting down. Moreover, the neighbors tell her that the owner of an inn where both couples had recently spent their holidays may soon have to close the business because of dramatic climate changes. For details, see the P2 in the Supplementary materials.

We developed the two stories to vary in the motif of threat while remaining comparable in terms of length, storyline, and the number of characters. Also, we informed respondents before they read the priming story that there would be a memory test at the end of the survey: in this way, we wanted them to focus on the stories and see which priming would affect the memory score more.

We validated the priming stories in a pilot study which was conducted at the beginning of March 2020 during the first lockdown, for which we recruited a total of 152 respondents (110 women and 42 men) aged at least 18 years old (women: M_age_ = 38.9, SD = 13.3, men: M_age_ = 40.1, SD = 12.2) (74 in experimental condition). We asked them to verbally rate the credibility of the assigned priming story (“How plausible was the story you read?”, “How engaging was the story?”, “Is there anything you would change to make the story more credible and impressive?”, “Which emotions did the story elicit in you?”). Furthermore, the pilot study contained items on basic sociodemographic status, the Ten-Item Personality Inventory (TIPI)^24^, the Trait Anxiety Inventory (TAI)^25^, the Three Domain Disgust Scale (TDDS)^4^, and a memory test, which assessed the memorability of our priming stories. We adapted the stories based on feedback from the respondents and made them more closely matched: we compared them sentence by sentence and adjusted them to match the corresponding sentence from the other story. For results of the pilot study, see S1 in the Supplementary materials.

### Measures

The sets of questionnaires were completed online through the Qualtrics survey platform. To start with, we asked some basic sociodemographic questions (such as age, gender, and education) and questions about long-term and current health issues (e.g. “How often do you suffer from headaches?” or “Do you use any medication prescribed by your family doctor?”). The health score was obtained from 9 items covering the incidence of health issues such as headaches, colds, or fatigue. The respondents rated each item on a verbally anchored 8-point scale. Response options were: “I don’t suffer from this issue at all” (scored as 0), “less than once a year”, “once a year”, “twice a year”, “every three months”, “once a month”, “once a week”, and “more often” (scored as 7). The final score could range from 0 to 63, with higher scores indicating a higher incidence of health issues. For a list of sociodemographic and health-related items, see S2 in the Supplementary materials. In the second round of the study, the sociodemographic questionnaire was considerably reduced. We focused on variables that may have changed and left aside variables such as height, weight, and occupation.

#### Trait anxiety

To assess trait anxiety, we used the 20-item Trait Anxiety Inventory (TAI), which explores stable anxiety traits^25^. It has been reported that anxiety traits affect disgust sensitivity^26^. Respondents were asked to assess how often they experienced feelings described in the questions. These self-reported questionnaires were rated on a 4-point scale. The possible replies for TAI were: “almost never” (scored as 1), “sometimes”, “often”, “almost always” (scored as 4). The scores for the test ranged between 20 and 80, with higher scores indicating higher anxiety levels.

#### Disgust measures

Disgust sensitivity was assessed by three different measures: (i) Three Domain Disgust Scale, (ii) Culpepper Disgust Image Set, and (iii) Body Odor Disgust Scale. A higher score in each measure indicated a higher level of disgust sensitivity.

The Three Domain Disgust Scale (TDDS)^4^ is a self-report questionnaire with 21 items consisting of three subscales (pathogen, moral, and sexual disgust), with 7 items in each subscale. The items were rated on a 7-point scale ranging from 0 (not disgusting at all) to 6 (extremely disgusting). The total score could range between 0 and 126 and each subscale between 0 and 42.

The Culpepper Disgust Image Set (C-DIS)^27^ contains 20 pathogen-salient and paired 20 pathogen-free images divided in four factors: hygiene issues, parasite/infection, food/environmental, and injury/viscera. The images were rated on a 7-point scale ranging from 0 (not at all disgusting) to 6 (extremely disgusting). Mean scores were computed for both groups of images. Finally, scores for images suggestive of pathogen-free conditions were subtracted from scores for the pathogen-salient images to obtain the final score.

The Body Odor Disgust Scale (BODS)^28^ is a self-report questionnaire with 12 items focused on body odors eliciting disgust. It is divided in two subscales depending on whether the source of odor is external (e.g. “You are sitting next to a stranger and notice that his feet are very smelly.”) or internal (e.g. “You are alone at home and notice that your feet are very smelly.”). Participants rated each item on a 5-point scale ranging from 1 (not disgusting at all) to 5 (extremely disgusting). The score for each domain was calculated as a mean value. Final scores could range between 1 and 5.

#### Memory test

At the end of the set of the questionnaires, participants completed a 10-item memory test with multiple-choice questions (five possible answers) with one correct answer based on the previous priming story, e.g. “What was the name of the man in the opening story?” or “How many days did they spend on their holidays?”. For the list of questions, see S3 in the Supplementary materials. The score could range between 0 and 10 points.

#### Covid-related items

After completing the memory test, respondents answered a question related to their Covid concerns (“Are you concerned about the coronavirus?”) and a question about avoidance of human contact (“Are you avoiding travel, public places, or places with a higher concentration of people because of the coronavirus?”). The items were rated on a scale ranging from 0 to 100, with higher values indicating a higher level of concern/avoidance.

### Statistical analyses

All statistical tests were performed using Jamovi v. 0.9.6.9 software (The jamovi project, 2021; https://www.jamovi.org). To assess possible relationships between the dependent variables (disgust scores [TDDS, C-DIS, BODS], and the memory test) and two independent variables, namely the priming condition and data collection point (in the spring of 2020 during the first lockdown or during temporary lifting of restrictions in summer 2021), we employed a repeated measure Analysis of Covariance (ANCOVA). In the follow-up analyses, we explored interindividual differences, including control for possible effects of sex, age, health status, and trait anxiety on the abovementioned dependent variables.

We excluded from the analysis incomplete questionnaires (with over one-fifth of each domain or questionnaire unanswered) and questionnaires with the same values across most items. Missing data in incomplete questionnaires (with less than a fifth of each domain) were supplemented by average scores for that domain. We also excluded respondents under 18 years of age and pregnant women because disgust sensitivity changes during pregnancy^29,30^.

## Results

### The effect of priming on perceived disgust during a period of high pathogen threat

The statistical analysis included 761 respondents, 379 of whom (293 women, 86 men) were exposed to the experimental priming and 382 individuals (293 women, 89 men) to the control priming. Table 1 shows descriptive statistics separately for women and men for parameters, such as age and disgust measures, collected during the period of high pathogen threat.

**Table 1:** Descriptive statistics for measures from respondents collected during the period of high pathogen threat in experimental and control priming conditions.

	Experimental priming (N = 379)				Control priming (N = 382)			
	Women (N = 293)		Men (N = 86)		Women (N = 293)		Men (N = 89)	
	M	SD	M	SD	M	SD	M	SD
Age	33.8	11.8	34.3	11.6	32.3	11.8	32.5	9.5
TDDS total	70.8	19.4	58.5	18.4	68.5	18.8	54.1	18.8
TDD-pathogen	23.8	7.6	21.1	7.4	23.1	7.4	19.0	7.0
TDD-moral	28.8	8.5	29.0	8.7	28.5	8.0	26.7	9.1
TDD-sexual	19.2	8.6	10.8	6.7	18.2	8.2	10.3	6.2
BODS-internal	2.9	0.9	2.8	0.8	3.0	0.9	2.6	0.9
BODS-external	4.0	0.7	3.8	0.7	4.0	0.7	3.6	0.9
C-DIS	3.8	0.8	3.7	0.9	3.9	0.8	3.8	0.8

**Figure 1:**
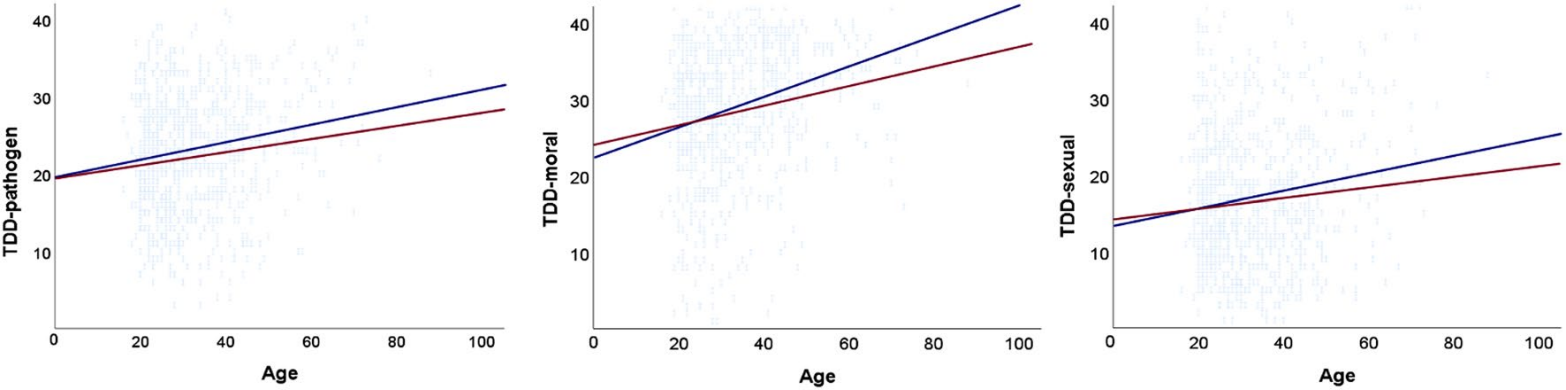
The association between respondents’ age and pathogen, moral, and sexual disgust scores in the experimental priming condition (blue line) and control priming condition (red line) during the period of high pathogen threat.

First, we compared the age between the experimental and control priming group and found that respondents in the experimental priming group were significantly older: t (759) = 1.85, p = 0.033, d = 0.134. To account for this confounding variable, we added age as a covariate to further analyses (see Fig. 1). Although women scored significantly higher on most disgust measures, we did not find a significant interaction between the priming condition and sex on disgust measures (see Table S4 in the Supplementary materials). The ANCOVA revealed a strong effect of age on all TDDS subscales scores, BODS-internal scores, and C-DIS scores but during the period of high pathogen threat, the effect of priming on TDDS total scores was not significant (see Table 2).

**Table 2:** Series of ANCOVA models testing the effect of priming and age on TDDS, BODS, and C-DIS during the period of high pathogen threat.

Variable		F	p	η^2^_p_
TDDS total score	Priming	2.68	0.102	0.004
	**Age**	**41.81**	** < 0.001**	**0.052**
TDD-pathogen	Priming	3.03	0.082	0.004
	**Age**	**18.10**	** < 0.001**	**0.023**
TDD-moral	Priming	0.76	0.382	0.001
	**Age**	**37.59**	** < 0.001**	**0.049**
TDD-sexual	Priming	1.44	0.231	0.002
	**Age**	**11.11**	** < 0.001**	**0.015**
BODS-internal	Priming	0.16	0.686	0.000
	**Age**	**5.35**	**0.021**	**0.007**
BODS-external	Priming	0.56	0.454	0.001
	Age	0.59	0.442	0.001
C-DIS	Priming	3.66	0.056	0.005
	**Age**	**6.36**	**0.012**	**0.009**

Statistically significant associations are marked in bold.

In line with preregistration, we analyzed individual TDDS subscales separately, because we expected the effect of priming to be specific to pathogen disgust and moral disgust. We found no differences between experimental and control priming conditions in pathogen, moral, or sexual disgust. Moreover, the effect of priming was bordering on a formal level of statistical significance in C-DIS score but in the opposite than predicted direction: C-DIS score was higher in the control condition than in the experimental condition, although the effect size was not large. Finally, we found no significant effect of priming on either the BODS-internal or BODS-external score.

For the descriptive statistics of participants who dropped out after the first round of data collection, see S5 in the Supplementary materials.

### Comparing BIS-related variables during the period of high and low pathogen threat

The statistical analysis included 224 respondents who completed the survey during both data collection rounds: 99 participants (80 women, 19 men) were exposed to the experimental priming and 125 participants (95 women, 30 men) to the control priming. Descriptive statistics for parameters age, health score, trait anxiety, disgust measures, memory test score and covid concerns and avoidance, are presented in Table 3.

**Table 3:** Descriptive statistics for measures from respondents participating during both the high and low pathogen threat period.

	High pathogen threat (N = 224)								Low pathogen threat (N = 224)							
	Experimental priming (N = 99)				Control priming (N = 125)				Experimental priming (N = 99)				Control priming (N = 125)			
	Women (N = 80)		Men (N = 19)		Women (N = 95)		Men (N = 30)		Women (N = 80)		Men (N = 19)		Women (N = 95)		Men (N = 30)	
	M	SD	M	SD	M	SD	M	SD	M	SD	M	SD	M	SD	M	SD
Age	32.4	11.8	33.9	14.2	31.1	10.2	31.2	11.3	33.6	11.8	35.1	14.1	32.4	10.2	32.3	11.3
Health score	28.7	8.4	19.7	9.04	27.2	9.3	23.2	9.4	27.6	8.3	19.2	8.2	25.9	9.2	21.8	10.0
TAI	47.3	11.8	41.1	10.9	46.4	11.7	40.8	9.6	46.6	10.9	41.4	9.1	45.9	11.5	40.3	10.9
TDDS total	70.6	18.0	56.5	15.9	65.4	19.1	52.0	17.9	63.5	21.5	53.8	20.4	61.5	19.2	50.2	19.3
TDD-pathogen	23.6	7.1	19.6	5.7	21.5	7.9	17.9	6.4	23.4	8.3	19.1	6.2	22.0	8.7	18.9	7.6
TDD-moral	28.0	9.7	26.9	11.0	26.4	10.1	25.5	11.6	22.3	11.4	24.7	13.3	22.3	10.9	21.9	11.7
TDD-sexual	19.1	8.2	10.0	4.7	17.5	8.2	8.6	5.3	17.4	8.6	10.0	5.7	17.2	7.6	9.4	5.8
BODS-internal	3.0	0.9	2.7	0.7	2.8	0.9	2.7	0.9	2.8	0.9	2.6	0.8	2.6	0.9	2.5	0.8
BODS-external	4.1	0.7	3.6	0.8	3.8	0.8	3.6	1.0	4.0	0.7	3.6	0.9	3.8	0.8	3.5	0.9
C-DIS	3.9	0.7	3.5	0.8	3.8	0.9	3.3	0.8	4.0	0.8	3.7	0.6	3.8	1.0	3.5	0.9
Memory score	7.2	1.8	6.1	1.8	7.4	1.8	7.0	1.8	7.0	1.9	6.2	1.7	7.3	1.9	7.1	1.4
Covid concerns	63.6	27.0	63.5	26.9	61.1	28.1	46.7	27.7	50.9	25.6	43.9	29.9	45.5	26.2	49.4	29.3
Covid avoidance	84.4	21.7	81.2	24.0	81.0	25.0	83.7	27.9	50.2	31.1	54.3	32.2	48.1	31.9	42.3	30.5

#### Disgust measures

A repeated measure ANCOVA with age, health score, and TAI score as covariates and the priming condition as a between-subject factor found that during the period of high pathogen threat, respondents did not score significantly higher in TDDS total score, pathogen, or sexual disgust scores than during the period of low pathogen threat. Respondents did, however, score significantly higher on the moral disgust subscale during the period of high pathogen threat. We found no statistically significant differences between the BODS subdomains scores and C-DIS scores between the two data collection points. Moreover, we found no significant interaction between the data collection point and the priming conditions. Age was positively associated with TDDS total score, moral disgust score, and C-DIS score. Furthermore, trait anxiety scores significantly positively affected TDDS total scores, pathogen and sexual disgust scores, BODS-internal scores, and C-DIS scores. For more detailed results, see Table 4. To investigate significant interactions, we further analyzed the association between TAI and BODS–internal separately for the period of high and low pathogen threat. We found a slightly stronger effect of TAI on BODS-internal during the period of low pathogen threat (r = 0.291, p =  < 0.001) than during the period of high pathogen threat (r = 0.156, p = 0.020), but the effect did not reach the threshold of statistical significance (z = 1.497, p = 0.067). A repeated measure ANCOVA with sex as an additional covariate can be found in the Supplementary materials (see Table S6).

**Table 4:** Series of the repeated measure ANCOVAs testing the effect of the period (high and low pathogen threat) and the priming condition on TDDS, BODS, and C-DIS scores controlling for age, health score and TAI.

Parameter name		F	p	η^2^_p_
TDDS total score	Period	2.995	0.085	0.013
	Priming	1.770	0.185	0.008
	Age	10.260	**0.002**	0.045
	TAI	9.140	**0.003**	0.040
	Health score	1.510	0.221	0.007
	Period * priming	2.787	0.096	0.013
	Period * age	0.064	0.801	0.000
	Period * TAI	0.068	0.794	0.000
	Period * health score	0.544	0.462	0.002
TDD-pathogen	Period	0.188	0.665	0.001
	Priming	2.400	0.123	0.011
	Age	1.830	0.178	0.008
	TAI	9.500	**0.002**	0.042
	Health score	1.290	0.258	0.006
	Period * priming	0.967	0.326	0.004
	Period * age	5.575	**0.019**	0.025
	Period * TAI	0.172	0.678	0.001
	Period * health score	0.798	0.373	0.004
TDD-moral	Period	6.667	**0.010**	0.030
	Priming	0.255	0.614	0.001
	Age	13.276	** < 0.001**	0.057
	TAI	1.330	0.250	0.006
	Health score	0.286	0.593	0.001
	Period * priming	1.087	0.298	0.005
	Period * age	4.292	**0.039**	0.019
	Period * TAI	0.082	0.775	0.000
	Period * health score	0.218	0.641	0.001
TDD-sexual	Period	1.262	0.262	0.006
	Priming	0.938	0.334	0.004
	Age	2.148	0.144	0.010
	TAI	6.748	**0.010**	0.030
	Health score	1.162	0.282	0.005
	Period * priming	1.262	0.262	0.006
	Period * age	0.008	0.931	0.000
	Period * TAI	0.457	0.500	0.002
	Period * health score	0.072	0.789	0.000
BODS-internal	Period	2.495	0.116	0.011
	Priming	2.160	0.143	0.010
	Age	1.630	0.202	0.007
	TAI	8.160	**0.005**	0.036
	Health score	1.400	0.237	0.006
	Period * priming	0.061	0.804	0.000
	Period * age	3.088	0.080	0.014
	Period * TAI	5.581	**0.019**	0.025
	Period * health score	0.038	0.845	0.000
BODS-external	Period	0.443	0.506	0.002
	Priming	3.100	0.080	0.014
	Age	1.510	0.221	0.007
	TAI	3.280	0.072	0.015
	Health score	1.820	0.179	0.008
	Period * priming	0.224	0.637	0.001
	Period * age	1.865	0.173	0.008
	Period * TAI	3.132	0.078	0.014
	Period * health score	0.313	0.576	0.001
C-DIS	Period	0.374	0.541	0.002
	Priming	1.430	0.233	0.007
	Age	5.280	**0.023**	0.026
	TAI	6.230	**0.013**	0.030
	Health score	1.170	0.280	0.006
	Period * priming	0.049	0.825	0.000
	Period * age	0.145	0.704	0.001
	Period * TAI	0.075	0.784	0.000
	Period * health score	0.644	0.423	0.003

Df is 1 for all variables and 219 for residuals.

Statistically significant associations are marked in bold.

We found no statistically significant differences between memory scores achieved during the periods of high vs. low pathogen threat (see Table S3 in the Supplementary materials). Our results also showed no differences in trait variables between the two time points (see Table S7 in the Supplementary materials).

#### Covid concerns

A repeated measure ANCOVA with age, health score, and TAI score as covariates and the priming condition as a between-subject factor found that during the period of high pathogen threat, respondents reported a statistically significantly higher level of avoidance of other people than during the period of low pathogen threat. We did not, however, find the same effect in relation to Covid concerns. TAI scores were positively associated with Covid concerns but not with Covid avoidance. Moreover, we found a significant interaction between Covid concerns and age, but the interaction differed between the rounds of data collection. For more detailed results, see Table 5. To investigate this significant interaction, we have analyzed the association between Covid concerns and age separately for the period of high and low pathogen threat. We found a stronger effect (z = 1.615, p = 0.05) of age on Covid concerns during the period of low pathogen threat (r = − 0.117, p = 0.086) than during the period of high pathogen threat (r = 0.038, p = 0.572).

**Table 5:** Series of the repeated measure ANCOVAs testing the effect of the period (high and low pathogen threat) and the priming condition on Covid concerns and avoidance controlling for age, health score and TAI.

Parameter name		F	p	η^2^_p_
Covid concerns	Period	0.073	0.787	0.000
	Priming	1.216	0.271	0.006
	Age	0.025	0.874	0.000
	TAI	6.814	**0.010**	0.032
	Health score	0.001	0.970	0.000
	Period * priming	0.009	0.924	0.000
	Period * age	8.045	**0.005**	0.037
	Period * TAI	0.605	0.437	0.003
	Period * health score	0.717	0.398	0.003
Covid avoidance	Period	16.459	** < 0.001**	0.075
	Priming	0.477	0.491	0.002
	Age	0.025	0.875	0.000
	TAI	1.910	0.168	0.009
	Health score	2.704	0.102	0.013
	Period * priming	1.019	0.314	0.005
	Period * age	0.558	0.456	0.003
	Period * TAI	1.855	0.175	0.009
	Period * health score	0.498	0.481	0.002

Df is 1 for all variables and 209 for residuals.

Statistically significant associations are marked in bold.

Moreover, we found positive associations between Covid concerns and majority of the disgust measures during the high pathogen threat period but not during the low pathogen threat period. Covid avoidance was negatively associated with BODS-internal and positively associated with TDDS total score during the period of high pathogen threat (see Table S8 in the Supplementary materials).

### Association between BIS-related variables and TAI and health scores

As predicted in the preregistration, we found positive associations between scores of trait anxiety and disgust measures during the high and low pathogen threat periods (see Table 6). These results indicate that respondents who scored higher on trait anxiety also scored higher on disgust. Similarly, we found positive associations between health scores and most of the disgust measures at both data collection points (see Table 6). Respondents who reported more health issues also scored higher on disgust.

**Table 6:** Partial Pearson’s correlation between disgust measures, TAI, and health scores controlled for age.

	High pathogen threat				Low pathogen threat			
	TAI score		Health score		TAI score		Health score	
	r	p	r	p	r	p	r	p
TDDS total score	0.244	< 0.001	0.172	0.005	0.235	< 0.001	0.191	0.002
TDD-pathogen	0.244	< 0.001	0.164	0.007	0.228	< 0.001	0.205	0.001
TDD-moral	0.109	0.052	0.073	0.138	0.087	0.097	0.032	0.319
TDD-sexual	0.204	0.001	0.154	0.011	0.227	< 0.001	0.214	< 0.001
BODS-internal	0.171	0.005	0.151	0.012	0.295	< 0.001	0.199	0.001
BODS-external	0.132	0.025	0.151	0.012	0.171	0.005	0.175	0.004
C-DIS	0.116	0.045	0.009	0.446	0.112	0.052	0.077	0.133

### Association between age and pathogen and moral disgust

We found a significant positive correlation between age and the moral disgust score (r = 0.165, p = 0.014) but not pathogen disgust score (r = 0.128, p = 0.055) or sexual disgust score (r = 0.068, p = 0.314) during the period of high pathogen threat. We also found a significant positive association between age and moral disgust score (r = 0.252, p =  < 0.001) but not pathogen disgust score (r = − 0.015, p = 0.826) and sexual disgust score (r = 0.063, p = 0.346) during the period of low pathogen threat.

**Figure 2:**
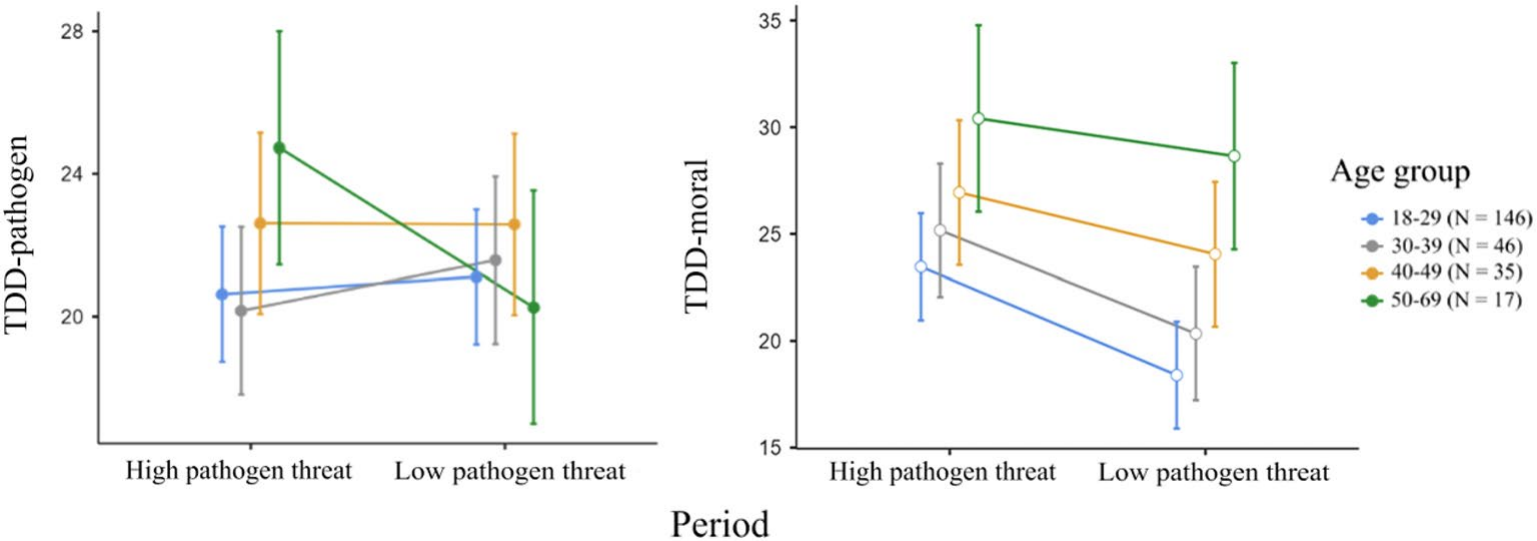
Comparison of age groups in pathogen and moral disgust score; error bars indicate 95% CI.

To further explore the effect of age on the pathogen and moral disgust scores, we split respondents in four age categories and repeated the analysis (a repeated measure ANCOVA) with age groups as a between-subject factor. We found a statistically significant interaction between data collection point (high vs. low pathogen threat) and age group in pathogen disgust score, F_3,220_ = 4.06, p = 0.008, η^2^_p_ = 0.052, but not in moral disgust score, F_3,220_ = 1.18, p = 0.316, η^2^_p_ = 0.016. Tukey’s HSD Test for multiple comparisons showed that the effect was strongly dependent on the group of 50–69 years old respondents regarding pathogen disgust because this was the only age category that differed between the data collection points (p_tukey_ = 0.05); see Fig. 2.

## Discussion

The main aim of the current study was to test whether the perception of high pathogen threat caused by the outbreak of the Covid-19 pandemic led to an increase in disgust sensitivity (especially pathogen and moral disgust). The perception of threat was induced by (i) a priming story and (ii) testing during a period of high pathogen threat (the beginning of the pandemic in the Czech Republic, the first lockdown) as compared to a period of low pathogen threat (14 months later, at a time when the Covid-19 incidence was relatively low). In contrast to our predictions, the results did not show heightened disgust in response to the experimental priming condition compared to the control priming condition at either data collection point. During the period of high pathogen threat respondents scored significantly higher on moral disgust but not on pathogen disgust than during the period of lower pathogen threat. Moreover, the effect was modulated by the age of respondents, being the highest among senior respondents.

Our findings regarding elevated moral disgust during periods of high pathogen threat are in line with a previous study which showed that individuals from nations with a higher pathogen burden tend to follow group norms more strictly^31^ and moral judging increases after exposure to disgusting odors or video clips^22^. Other studies, however, failed to replicate these results and the overall picture is thus rather ambivalent^32^. In our study, we also found a significant interaction between the data collection point and the age of respondents, with the highest moral disgust in the oldest age category. This might be due to respondents’ awareness of Covid-19 posing increased risk of serious illness in older persons^33^. Therefore, they may perceive any violation of rules as more disgusting.

Our finding of no difference in pathogen disgust between the periods of low and high pathogen threat conflicts with the results of several other studies which did report higher scores on self-reported disgust scale^14^ or higher disgust sensitivity using a naturalistic measure^12^ during a period of high pathogen threat. In the latter study, however, the difference in the TDDS pathogen domain between groups assessed before and during a pandemic “wave” was also not formally statistically significant. The disgust measures based on statements were originally designed for assessing interindividual variation, so it is possible that they are ill-suited for detection of intraindividual variation, such as one may find during periods of immune vulnerability^34^. The absence of a difference in pathogen disgust between the two data collection points (during a lockdown and after restrictions were lifted) may be due to the use of the TDDS questionnaire, which seems to be less sensitive to environment-related fluctuations in pathogen avoidance. Interestingly, we also failed to find the difference using the C-DIS, which includes images of disease cues and is considered to be a more sensitive measure of variation in pathogen disgust.

It should be noted that the studies reviewed above compared two different groups of respondents at different time points (e.g. before and during  pandemic). It is thus possible that in our study, pathogen disgust measured during a period of low pathogen threat (after most restrictions were lifted) remained elevated and was as high as at the beginning of the pandemic. It is perhaps because the threat was perceived as not being quite over yet. Nevertheless, the difference in incidence of Covid-19 infections (44 per 100,000 persons and 25 per 100,000 persons) between the two periods clearly shows that our decision to treat the two time points as periods high and low pathogen threat was justified (incidence is almost doubled), although the months that followed showed that case numbers could rise to values^35^ we could not anticipate when we designed the study in March 2020, at the outset of the pandemic which lasted globally for over two years. Nevertheless, one may argue that the two periods did not differ in overall pathogen threat as we expected, because we took into account only the incidence of Covid-19 cases but not of other infectious agents. Data from the National Institute of Public Health in Czech Republic (https://szu.cz/publikace/data/akutni-respiracni-infekce-chripka/) show that at the beginning of the Covid pandemic, influenza as a viral respiratory tract infection causing morbidity and mortality worldwide was still active, although its incidence was slowly decreasing. In early April 2020, the incidence of influenza was rather low and hotspots local, while Covid-19 started to be more prominent. The entire season of 2020/2021 was significantly influenced by Covid-19 and associated restrictions, which caused a milder course of the seasonal influenza epidemic. Overall data show that the spread of influenza was during the Covid-19 pandemic drastically reduced compared to previous years^36^. Such massive reduction in case numbers has been attributed primarily to mask wearing, which was in the Czech Republic obligatory during the period of high pathogen threat, while lower levels of contact among people also played a significant role. Importantly, objective measures of high pathogen threat (e.g. incidence of new cases) need not have directly translated into subjectively perceived threat level and consequently to higher levels of disgust. It is, for instance, possible that during the lockdown, people were exposed to markers of infection—such as coughing—to a smaller extent because most of the time they stayed at home. Moreover, a substantial percentage of people who become infected with Covid-19 remain asymptomatic and many transmission routes do not provide sufficient cues^37^. This might help explain why we did not find any elevation of pathogen disgust. Nevertheless, we do not think this can entirely explain the null finding because the respondents did report being more concerned about Covid-19 during the period of high pathogen threat and they also reported that they avoid public spaces and travel.

The lack of difference in pathogen disgust between the periods of high and low pathogen threat might be also caused by the selection bias. Specifically, respondents who showed elevated disgust levels may have been more likely to participate in the second round of data collection. But this explanation seems unlikely. We compared individuals who took part only in the first round and those who participated in both rounds and found that respondents who took part in both rounds of data collection scored lower on pathogen and moral disgust than those who took part only in the first round. This is the opposite of what one would expect in a case of selection bias for null findings.

We found no effect of the priming condition on disgust sensitivity, which was expected in the preregistration. We predicted that an elevation of disgust levels during the period of high pathogen threat would preclude further efficiency of the experimental priming. During the period of low pathogen threat, on the other hand, we expected to see an increase in disgust sensitivity after exposure to the experimental priming. The stories were carefully designed during the first wave of the Covid-19 pandemic in spring 2020 and provisionally validated based on a data from the pilot study. The use of vignettes is a common tool used in psychological research^38^ and vignettes are also widely employed in a research on sexual and moral disgust^17–19^. Nevertheless, one might argue that pathogen disgust was selected primarily to respond to perceptual cues of infection (e.g. the smell of wounds), while indirect stimuli—such as the story we used—need not be an efficient disgust trigger. On the other hand, there is robust evidence to the effect that affective states, including disgust, can be easily induced by symbolic communication either in spoken or written form^39^. Furthermore, disgusting stories boost item recall and recognition^40^, although our results did not show any difference in the memory test results between the experimental and control priming story.

We also found that older respondents showed overall higher pathogen and moral disgust than younger respondents did at both data collection points, which is partly in line with our preregistered hypotheses and our pilot study. We assumed that older persons are more vulnerable to diseases because the functionality of their physiological immune system is declining^41^. Their disgust sensitivity and functionality of the BIS should therefore be elevated to compensate for this possible deficiency^42^. This assumption finds support in a recent study which found that germ aversion increases with age^15^. Our results also support the notion of BIS functioning as a compensatory mechanism because the increase in pathogen disgust at a time of high pathogen threat was restricted to the oldest age group (50–69 years). On the other hand, it has also been predicted that disgust sensitivity should decline with declining reproductive potential (i.e. with age). For example, Curtis et al.^6^ found that younger participants were more disgusted by pathogen-salient pictures than older participants were. Several other studies similarly reported a negative correlation between age and disgust levels^7,43,44^, but it should be noted that most of those studies did not involve participants over fifty, when further changes may occur (as shown by our data).

Our results also did not show any significant changes in trait anxiety and general health status between the two data collection points, which is in line with the preregistered hypotheses. These variables are considered stable. They can, however, influence the disgust scores: for instance, scores from trait anxiety and scores from the health status questionnaire were positively correlated with most of the disgust scores. These results are thus in line with the results of other studies, which also found a positive association between disgust sensitivity, health, and general anxiety scores^45,46^.

### Limitations

The current study relied on self-report measures. This can be considered a limitation because respondents need not be aware of changes in their disgust sensitivity, but they may still occur. Nevertheless, respondents also reported a smaller tendency to meet other people and slightly higher levels of concern about the coronavirus during the high-risk period. This indicates avoidance of the obvious routes by which Covid-19 spreads, but it may not be directly linked to pathogen disgust sensitivity.

Inspection of the age distribution of our sample shows that a rather small part of participants was over fifty years of age and majority of the sample was composed of young adults. Nevertheless, most of the participants samples in previous studies consist of young people. Clearly, more studies involving older participants are needed to better understand the age-dependent dynamics of disgust sensitivity.

Although we also carefully designed and provisionally validated the priming vignettes, their content may have been perceived as less relevant during the period of low pathogen threat, in summer 2021, which may have limited their efficiency. It points to the variable dynamics of the Covid-19 pandemic and shows that scholars should differentiate between the individual waves of the pandemic^47,48^.

## Conclusions

Our findings based on a within-subject study show heightened moral disgust, but not increased pathogen disgust, during the first outbreak of the Covid-19 pandemic in the Czech Republic. Moreover, respondents’ age positively correlated with both pathogen and moral disgust, supporting a hypothesis according to which the BIS may compensate for age-related decline in the functionality of the physiological immune system, which can come to the fore especially during a period of increased pathogen threat. Our study indicates that variations in pathogen disgust sensitivity—at least as measured by the TDDS—may depend more on age or trait anxiety as more stable characteristics and less on the relatively fast changing situation regarding current threats to health.

Our study also highlights the importance of differentiating between the objective level of threat and subjectively perceived threat. Finally, the findings indicate that the cognitive and perceptual components of BIS need not be always aligned: during the lockdown, the level of stimulation by pathogen cues (the perceptual component) might have been low, while concerns about infection (the cognitive component) were elevated. Future studies should therefore consider using a combination of behavioral tests and self-reports to capture various components of the BIS.

## Supplementary Information

Supplementary Information.

## Data Availability

The data associated with this research are available at (https://osf.io/sy6wn/).
