# Supplementary material for: Pandemic elevates sensitivity to moral disgust but not pathogen disgust
Source: Sci Rep. 2023 May 22;13:8206. doi: 10.1038/s41598-023-35375-2 (PMC10203323; doi:10.1038/s41598-023-35375-2)
Supplement: Supplementary file 1 — Supplementary Information. [file 41598_2023_35375_MOESM1_ESM.docx]

**PANDEMIC ELEVATES SENSITIVITY TO MORAL DISGUST BUT NOT PATHOGEN DISGUST**

Dagmar Schwambergová^1,2^, Šárka Kaňková^3^, Jitka Třebická Fialová^1^, Jana Hlaváčová^3^, and Jan Havlíček^1,2^

^1^ Department of Zoology, Faculty of Science, Charles University, Viničná 7, 128 44 Prague 2, Czech Republic

^2^ National Institute of Mental Health, Topolová 748, 250 67 Klecany, Czech Republic

^3^ Department of Philosophy and History of Science, Faculty of Science, Charles University, Viničná 7, 128 44 Prague 2, Czech Republic

* Corresponding author:

Tel.: +420 221951853, fax: +420 221951125

E-mail: schwambd@natur.cuni.cz

Department of Zoology, Faculty of Science, Charles University, Viničná 7, 128 44 Prague 2, Czech Republic

**Supplemental Material**

**P1:** EXPERIMENTAL PRIMING

*Original (Czech):* Na únorové lyžování ve středisku Tarvisio v oblasti Friuli na severu Itálie pan Vladislav s manželkou Janou dlouho nezapomenou. Po vydařené dovolené se oba vrátili do Čech plni vzpomínek a pozitivních zážitků. Jejich obyčejný život se ale po několika dnech změnil takřka k nepoznání. Pan Vladislav se začal cítit slabý, jako by se o něj pokoušela chřipka. K horečkám se však brzy přidal i nezvykle silný kašel, a tak se ho manželka rozhodla odvézt do nemocnice, kde mu byl proveden výtěr z úst a nosu. Diagnóza ze Státního zdravotního ústavu přišla záhy. Nakazil se novým typem koronaviru a vypuklo u něj onemocnění COVID-19. Protože jeho nemoc probíhala bez vážnějších komplikací, byl poslán vyležet se domů. Paní Jana sice žádné příznaky neměla, přesto jí byla nařízena dvoutýdenní karanténa. Jenže nutně potřebovala vzniklou situaci s někým probrat. Zašla proto na kávu k sousedům - staršímu manželskému páru. Po dvou týdnech se nákaza projevila i u sousedky, která navíc trpěla silným astmatem. Hospitalizace na infekčním oddělení v Motole na sebe nenechala dlouho čekat. Její stav se však rychle zhoršoval a s těžkým zápalem plic musela být napojena na plicní ventilaci.

*Translation:* Mr. Vladislav and his wife Jana will remember their February skiing holidays at the Tarvisio resort, the Friuli region, northern Italy. After a successful holiday, they both returned to Czechia full of memories and positive experiences. But after a few days, their ordinary lives changed completely. Mr. Vladislav started to feel weak as if he had caught the flu. However, an unusually strong cough soon added to the already existing fever, so his wife decided to take him to the hospital, where he had a nasal swab. The diagnosis from the State Health Department arrived soon after. He had been diagnosed with a new type of coronavirus and had an outbreak of the disease called COVID-19. Because his illness progressed without serious complications, he was sent home from the hospital. Although Mrs. Jana had no symptoms, she was ordered to stay in a quarantine for two upcoming weeks. But she had strong urges to discuss with someone what has happened lately in her life. So she paid a visit to her neighbours - an older couple. After two weeks, her neighbour, who suffered from severe asthma, also got infected with COVID-19. Shortly after, she had to be hospitalized in the infectious diseases department in Motol hospital. However, her condition worsened rapidly and she had to be connected to pulmonary ventilation with severe pneumonia.

**P2:** CONTROL PRIMING

*Original (Czech):* Na únorovou dovolenou, skialpinistický přechod ledovce Okjokull v oblasti Faxaflói na jihovýchodě Islandu, pan Vladislav s manželkou Janou dlouho nezapomenou. Hned po příjezdu na místo totiž zjistili, že v celé oblasti není sníh. Nemile překvapená manželka volala hned druhý den na horskou službu a meteorologickou stanici. Snažila se získat přesné informace o předpovědi počasí a zejména možnostech sněžení v následujících dnech. Meteorologové předpovídali husté sněžení během noci. Po dvou dnech se manželé opravdu probudili do bílého rána, jenže teploty se během dne vyšplhaly k 9 až 11 °C, a sníh tak neměl dlouhého trvání. Původně týdenní dovolenou proto ukončili již čtvrtý den a odjeli zpátky domů. Paní Jana nutně potřebovala vzniklou situaci s někým probrat. Zašla proto na kávu k sousedům - staršímu manželskému páru. Ti jí k jejímu překvapení sdělili, že před dvaceti a patnácti lety na tom samém místě provedli dva nezapomenutelné přechody ledovce. Když se tam však v loňském roce vrátili, sníh tam již nebyl a ledovec se k nepoznání zmenšil. Navíc jí prozradili, že jim nedávno psal majitel penzionu Selfoss, kde oba manželské páry trávili své dovolené, že kvůli změně klimatu krachuje a bude muset penzion zavřít.

*Translation:* Mr. Vladislav and his wife Jana will remember their February holidays, the ski mountaineering trek of the Okjokull glacier in the Faxaflói area in the southeast of Iceland. Immediately after arriving, they found out that there was no snow in the whole area. The disappointingly surprised wife called the mountain rescue and the meteorological station the very next day. She tried to obtain accurate information about the weather forecast and especially the possibilities of snowfall in the following days. Meteorologists predicted heavy snowfall during the night. After two days, the couple really woke up to a white morning, but the temperatures climbed to 9 - 11 ° C during the day, and the snow didn’t last long. Therefore, they terminated their originally week-long holiday on the fourth day and went back home. Mrs. Jana urgently needed to discuss the situation with someone. So she paid a visit to her neighbours - an older couple. To her surprise, they told her that twenty and fifteen years ago they had made two unforgettable glacier crossings at the same place. However, when they returned there last year, the snow was no longer there and the glacier had shrunk beyond recognition. In addition, they told her that the Selfoss guesthouse owner, where both couples had spent their holidays recently, wrote to them that he was going bankrupt due to the climate change and would have to close the guesthouse.

**S1:** PILOT STUDY

The statistical analysis included 152 respondents, of whom 74 were exposed to the experimental priming and 78 control priming. Descriptive statistics for parameters, such as age, disgust, anxiety, and stress measures and memory score collected in pilot study, are presented in Table S1a.

|  | **Experimental priming (N = 74)** | | | **Control priming (N = 78)** | | |
| --- | --- | --- | --- | --- | --- | --- |
|  | **M** | **SD** | **Range (min – max)** | **M** | **SD** | **Range (min – max)** |
| **Age** | 41.4 | 14.3 | 20 – 72 | 37.1 | 11.5 | 18 – 70 |
| **TDDS total** | 74.1 | 18.4 | 43 – 125 | 70.6 | 19.9 | 15 – 116 |
| **TDD-pathogen** | 25.4 | 8.24 | 10 – 41 | 24.4 | 8.00 | 5 – 40 |
| **TDD-moral** | 30.5 | 7.61 | 0 – 42 | 28.6 | 8.93 | 0 – 42 |
| **TDD-sexual** | 18.2 | 9.91 | 0 – 42 | 17.5 | 9.98 | 0 – 40 |
| **SAI** | 39.0 | 13.9 | 20 – 73.3 | 37.7 | 11.5 | 20 – 70 |
| **PSS** | 18.1 | 4.80 | 9 – 33 | 18.3 | 5.67 | 6 – 33 |
| **Memory score** | 6.65 | 2.09 | 1 – 10 | 6.46 | 1.98 | 1 – 10 |

*Table S1a: Descriptive statistics for measures from respondents participating in pilot study.*

An Analysis of Covariance (ANCOVA) revealed a strong influence of age on TDDS total score, pathogen and sexual TDDS, and memory score. However, the model showed that the effect of priming on all analysed BIS variables was not significant in the pilot study, see Table S1b.

| **Variable** | | **F** | **p** | **η^2^_p_** |
| --- | --- | --- | --- | --- |
| **TDDS total score** | Priming | 0.472 | 0.493 | 0.003 |
|  | **Age** | **7.283** | **0.008** | **0.047** |
| **TDD-pathogen** | Priming | 0.138 | 0.711 | 0.001 |
|  | **Age** | **5.39** | **0.035** | **0.035** |
| **TDD-moral** | Priming | 1.20 | 0.275 | 0.008 |
|  | Age | 2.26 | 0.135 | 0.015 |
| **TDD-sexual** | Priming | 0.007 | 0.931 | 0.000 |
|  | **Age** | **3.983** | **0.048** | **0.026** |
| **SAI** | Priming | 1.377 | 0.242 | 0.009 |
|  | Age | 0.014 | 0.907 | 0.000 |
| **PSS** | Priming | 3.20e-4 | 0.986 | 0.000 |
|  | Age | 3.16 | 0.078 | 0.021 |
| **Memory score** | Priming | 1.99 | 0.161 | 0.013 |
|  | **Age** | **23.87** | **< 0.001** | **0.138** |

*Table S1b: Series of ANCOVA models testing the effect of priming and age on TDDS, SAI, PSS and memory score . Statistically significant associations are marked in bold.*

**S2:** SOCIODEMOGRAPHIC AND HEALTH-RELATED ITEMS

| Sociodemographic questions: | | |
| --- | --- | --- |
|  |  |  |
| 1. What is your gender? | | |
| 1. What is your age (in years)? | | |
| 1. What is your height (in cm)? | | |
| 1. What is your weight (in kg)? | | |
| 1. How many residents had the village/city where you had been living until 15 years of age? | | |
| 1. How many residents has the village/city you are living in now? | | |
| 1. What is your current occupation? (Specify the position.) | | |
| 1. What is the highest level of education you have completed? | | |
| 1. What is the current income of your household (in CZK)? | | |
| 1. What is your family status? | | |
| 1. Are you currently pregnant? | | |
| 1. How many biological children do you have? | | |
| 1. How many members do your household have? | | |
|  |  |  |
| Health-related questions: | | |
|  |  |  |
| 1. How are you feeling physically today? | | |
| 1. How are you feeling mentally today? | | |
| 1. How have you been feeling physically in the last year? | | |
| 1. How have you been feeling mentally in the last year? | | |
| 1. Do you use any medication prescribed by the doctor? | | |
| 1. Do you use any other medication? | | |
| 1. How many times did you use antibiotics in the last 365 days? | | |
| 1. Have you had any health issues in the last week? | | |
| 1. Do you have any chronical long-term health problems? | | |
| 1. Do you suffer from an insufficient immunity? | | |
| 1. How often do you suffer from headaches? | | |
| 1. How often do you suffer from cold? | | |
| 1. How often do you suffer from nausea? | | |
| 1. How often do you suffer from sore throat and cough? | | |
| 1. How often do you suffer from allergies? | | |
| 1. How often do you suffer from sleeping issues? | | |
| 1. How often do you suffer from urinary tract inflammation? | | |
| 1. How often do you suffer from increased fatigue? | | |
| 1. How often do you suffer from common bacterial and viral infections? | | |

*Table S2: Overview of questions from sociodemographic and health-related questionnaire.*

**S3:** MEMORY TEST – ITEMS AND RESULTS

| Questions in memory test – experimental priming: | | |
| --- | --- | --- |
|  |  |  |
| 1. What was the name of the man in the opening story? | | |
| 1. When was he on holidays? | | |
| 1. Where was he on holidays (place, region, country)? | | |
| 1. What kind of symptoms did he have? | | |
| 1. Who took the man to the hospital? | | |
| 1. Where did the test for COVID-19 take place? | | |
| 1. Who was in contact with the man? | | |
| 1. When did the neighbour start having symptoms? | | |
| 1. What kind of health complications the neighbour’s had? | | |
| 1. Where was the neighbour hospitalized? | | |
|  |  |  |
| Questions in memory test – control priming: | | |
|  |  |  |
| 1. What was the name of the man in the opening story? | | |
| 1. When was he on holidays? | | |
| 1. Where was he on holidays (place, region, country)? | | |
| 1. With who did his wife communicate about the weather? | | |
| 1. How many days they spent on their holidays? | | |
| 1. When and after how many days did the snow fall? | | |
| 1. What were the daily temperatures? | | |
| 1. Where did they stay during their holidays? | | |
| 1. Who did Mrs. Jana meet after the holidays? | | |
| 1. When did her acquaintances cross the glacier? | | |

*Table S3a: Overview of questions from memory tests.*

**Results – Memory test**

We did not find any statistically significant effect of the period on overall memory score, however, the score was negatively affected by the age of respondents; for detailed results, see Table S3b.

| **Parameter name** | | **F** | **p** | **η^2^_p_** |
| --- | --- | --- | --- | --- |
| **Memory score** | Period | 1.214 | 0.272 | 0.006 |
|  | Priming | 1.946 | 0.164 | 0.009 |
|  | Age | 21.963 | **< 0.001** | 0.091 |
|  | TAI | 1.932 | 0.166 | 0.009 |
|  | Health score | 0.003 | 0.956 | 0.000 |
|  | Period * priming | 0.010 | 0.917 | 0.000 |
|  | Period * age | 0.382 | 0.537 | 0.002 |
|  | Period * TAI | 1.618 | 0.205 | 0.007 |
|  | Period * health score | 0.061 | 0.805 | 0.000 |

*Table S3b:* *The repeated measure ANCOVA testing effect of age, health score, TAI, and priming condition on final memory score. Df is equal to 1 for all variables and 219 for residuals. Statistically significant associations are marked in bold.*

**S4:** SEX DIFFERENCES IN BIS VARIABLES IN HIGH PATHOGEN THREAT PERIOD

We found significant differences between female and male respondents in pathogen, sexual and the total TDDS, and in both BODS domains, but not in moral TDDS and C-DIS scores. Female respondents scored higher in these disgust measures, see S5b.

| **Variable** | | **F** | **p** | **η^2^_p_** |
| --- | --- | --- | --- | --- |
| **TDDS total score** | Priming | 2.910 | 0.088 | 0.004 |
|  | **Age** | **47.05** | **< 0.001** | **0.059** |
|  | **Sex** | **72.03** | **< 0.001** | **0.087** |
|  | Priming * Sex | 0.354 | 0.552 | 0.000 |
| **TDD-pathogen** | Priming | 3.95 | 0.057 | 0.005 |
|  | **Age** | **19.32** | **< 0.001** | **0.025** |
|  | **Sex** | **29.29** | **< 0.001** | **0.037** |
|  | Priming * Sex | 1.03 | 0.309 | 0.001 |
| **TDD-moral** | Priming | 2.03 | 0.155 | 0.003 |
|  | **Age** | **37.61** | **< 0.001** | **0.049** |
|  | Sex | 1.28 | 0.258 | 0.002 |
|  | Priming * Sex | 1.65 | 0.199 | 0.002 |
| **TDD-sexual** | Priming | 0.676 | 0.411 | 0.001 |
|  | **Age** | **13.66** | **< 0.001** | **0.018** |
|  | **Sex** | **134.45** | **< 0.001** | **0.153** |
|  | Priming * Sex | 0.149 | 0.700 | 0.000 |
| **BODS - internal** | Priming | 1.08 | 0.300 | 0.001 |
|  | **Age** | **5.60** | **0.018** | **0.007** |
|  | **Sex** | **12.07** | **< 0.001** | **0.016** |
|  | Priming * Sex | 1.76 | 0.185 | 0.002 |
| **BODS - external** | Priming | 1.87 | 0.172 | 0.002 |
|  | Age | 0.674 | 0.412 | 0.001 |
|  | **Sex** | **17.516** | **< 0.001** | **0.023** |
|  | Priming * Sex | 1.977 | 0.160 | 0.003 |
| **C-DIS** | Priming | 2.156 | 0.142 | 0.003 |
|  | **Age** | **6.41** | **0.012** | **0.009** |
|  | Sex | 1.145 | 0.285 | 0.002 |
|  | Priming * Sex | 0.118 | 0.732 | 0.000 |

*Table S4: Series of ANCOVA models testing the effect of priming, sex and age on TDDS, BODS, and C-DIS . Statistically significant associations are marked in bold.*

**S5:** DESTRIPTIVE STATISTICS OF RESPONDENTS WHO DROPPED OUT

The statistical analysis included 537 respondents, of whom 280 were exposed to the experimental priming and 257 control priming. Descriptive statistics separately for women and men for parameters, such as age, disgust, anxiety, and memory score, are presented in Table S5a.

|  | **Experimental priming (N = 280)** | | | | | **Control priming (N = 257)** | | | | |
| --- | --- | --- | --- | --- | --- | --- | --- | --- | --- | --- |
|  | **Women (N = 213)** | | **Men (N = 67)** | | | **Women (N = 197)** | | **Men (N = 60)** | | |
|  | **M** | **SD** | | **M** | **SD** | **M** | **SD** | | **M** | **SD** |
| **Age** | 34.3 | 11.7 | | 34.4 | 10.8 | 32.9 | 12.6 | | 33.3 | 8.54 |
| **Health score** | 25.8 | 9.04 | | 20.0 | 8.45 | 26.0 | 9.62 | | 22.7 | 9.41 |
| **TAI** | 45.7 | 5.52 | | 43.3 | 4.26 | 46.1 | 5.55 | | 43.5 | 4.33 |
| **TDDS total** | 71.0 | 20.0 | | 59.1 | 18.5 | 69.9 | 18.5 | | 55.6 | 19.2 |
| **TDD-pathogen** | 24.0 | 7.86 | | 21.5 | 7.78 | 23.6 | 7.43 | | 19.6 | 7.23 |
| **TDD-moral** | 28.8 | 8.61 | | 28.8 | 9.32 | 28.9 | 7.39 | | 27.2 | 8.27 |
| **TDD-sexual** | 19.4 | 8.84 | | 11.0 | 7.20 | 18.6 | 8.22 | | 11.1 | 6.63 |
| **BODS-internal** | 2.98 | 0.92 | | 2.86 | 0.89 | 3.07 | 0.92 | | 2.59 | 0.91 |
| **BODS-external** | 3.97 | 0.76 | | 3.87 | 0.75 | 4.05 | 0.76 | | 3.64 | 0.84 |
| **C-DIS** | 3.98 | 0.74 | | 3.74 | 0.92 | 3.99 | 0.89 | | 3.46 | 0.92 |
| **Memory score** | 7.09 | 1.87 | | 6.51 | 1.81 | 7.22 | 1.98 | | 6.71 | 2.02 |

*Table S5a: Descriptive statistics for measures from respondents participating in the high pathogen threat period, but not in the low pathogen threat period.*

An Analysis of Covariance (ANCOVA) revealed a strong influence of age on all domains of TDDS, BODS – internal, C-DIS, and memory score. Moreover, we found a difference in pathogen and moral TDDS in participants who took part only in high pathogen threat period and participants who took part in both periods, but these effects were negligible, see Table S5b. These results show that respondents scoring higher in pathogen and moral disgust during the first data collection period were more likely to drop out.

| **Variable** | | **F** | **p** | **η^2^_p_** |
| --- | --- | --- | --- | --- |
| **TDDS total score** | Group | 1.66 | 0.198 | 0.002 |
|  | **Age** | **40.98** | **<0.001** | **0.051** |
| **TDD-pathogen** | **Group** | **4.38** | **0.037** | **0.006** |
|  | **Age** | **17.71** | **<0.001** | **0.023** |
| **TDD-moral** | **Group** | **4.13** | **0.042** | **0.006** |
|  | **Age** | **34.74** | **<0.001** | **0.045** |
| **TDD-sexual** | Group | 1.44 | 0.230 | 0.002 |
|  | **Age** | **10.11** | **0.002** | **0.013** |
| **BODS - internal** | Group | 0.354 | 0.552 | 0.000 |
|  | **Age** | **5.290** | **0.022** | **0.007** |
| **BODS - external** | Group | 0.263 | 0.608 | 0.000 |
|  | Age | 0.657 | 0.418 | 0.001 |
| **C-DIS** | Group | 1.93 | 0.165 | 0.003 |
|  | **Age** | **4.17** | **0.042** | **0.006** |
| **Memory score** | Group | 0.305 | 0.581 | 0.000 |
|  | **Age** | **73.806** | **<0.001** | **0.089** |

*Table S5b: Series of ANCOVA models testing the effect of group (participants who took part in the study only during the first wave or both waves) and age on TDDS, BODS, C-DIS and memory score. Statistically significant associations are marked in bold.*

**S6:** SEX DIFFERENCES IN DISGUST MEASURES AND TRAIT ANXIETY BETWEEN THE PERIODS OF HIGH PATHOGEN THREAT AND LOW PATHOGEN THREAT

We found significant differences between female and male respondents in pathogen, sexual and the total TDDS, and in both BODS-external and C-DIS scores, but not in moral TDDS and BODS-internal. However, there was no significant interaction between sex and period and/or priming, see Table S6a.

| **Parameter name** | | **F** | **p** | **η^2^_p_** |
| --- | --- | --- | --- | --- |
| **TDDS total score** | Period | 3.391 | 0.067 | 0.015 |
|  | Priming | 1.111 | 0.293 | 0.005 |
|  | Age | 10.432 | **0.001** | 0.046 |
|  | Sex | 11.04 | **0.001** | 0.048 |
|  | TAI | 7.534 | **0.007** | 0.034 |
|  | Health score | 0.337 | 0.562 | 0.002 |
|  | Priming * sex | 0.017 | 0.898 | 0.000 |
|  | Period * priming | 0.814 | 0.368 | 0.004 |
|  | Period * age | 0.068 | 0.794 | 0.000 |
|  | Period * TAI | 0.147 | 0.702 | 0.001 |
|  | Period * health score | 1.271 | 0.261 | 0.006 |
|  | Period * sex | 3.227 | 0.074 | 0.015 |
|  | Period * priming * sex | 0.476 | 0.491 | 0.002 |
| **TDD-pathogen** | Period | 0.145 | 0.704 | 0.001 |
|  | Priming | 1.049 | 0.307 | 0.005 |
|  | Age | 1.794 | 0.182 | 0.008 |
|  | Sex | 5.155 | **0.024** | 0.023 |
|  | TAI | 8.351 | **0.004** | 0.037 |
|  | Health score | 0.417 | 0.519 | 0.002 |
|  | Priming * sex | 0.079 | 0.778 | 0.000 |
|  | Period * priming | 0.799 | 0.372 | 0.004 |
|  | Period * age | 5.479 | **0.020** | 0.025 |
|  | Period * TAI | 0.232 | 0.630 | 0.001 |
|  | Period * health score | 0.931 | 0.336 | 0.004 |
|  | Period * sex | 0.341 | 0.560 | 0.002 |
|  | Period * priming * sex | 0.042 | 0.837 | 0.000 |
| **TDD-moral** | Period | 7.057 | **0.008** | 0.031 |
|  | Priming | 0.439 | 0.508 | 0.002 |
|  | Age | 13.134 | **< 0.001** | 0.057 |
|  | Sex | 0.135 | 0.714 | 0.001 |
|  | TAI | 1.317 | 0.252 | 0.006 |
|  | Health score | 0.389 | 0.533 | 0.002 |
|  | Priming * sex | 0.175 | 0.676 | 0.001 |
|  | Period * priming | 0.032 | 0.859 | 0.000 |
|  | Period * age | 4.291 | **0.039** | 0.019 |
|  | Period * TAI | 0.047 | 0.828 | 0.000 |
|  | Period * health score | 0.699 | 0.404 | 0.003 |
|  | Period * sex | 2.269 | 0.133 | 0.010 |
|  | Period * priming * sex | 1.165 | 0.282 | 0.005 |
| **TDD-sexual** | Period | 1.551 | 0.214 | 0.007 |
|  | Priming | 0.393 | 0.531 | 0.002 |
|  | Age | 2.293 | 0.131 | 0.010 |
|  | Sex | 39.221 | **< 0.001** | 0.153 |
|  | TAI | 4.856 | **0.029** | 0.022 |
|  | Health score | 0.008 | 0.931 | 0.000 |
|  | Priming * sex | 2.16e-4 | 0.988 | 0.000 |
|  | Period * priming | 1.298 | 0.256 | 0.006 |
|  | Period * age | 0.010 | 0.920 | 0.000 |
|  | Period * TAI | 0.672 | 0.413 | 0.003 |
|  | Period * health score | 0.425 | 0.515 | 0.002 |
|  | Period * sex | 3.325 | 0.070 | 0.015 |
|  | Period * priming * sex | 0.166 | 0.684 | 0.001 |
| **BODS-internal** | Period | 2.652 | 0.105 | 0.012 |
|  | Priming | 1.027 | 0.312 | 0.005 |
|  | Age | 1.619 | 0.205 | 0.007 |
|  | Sex | 0.296 | 0.587 | 0.001 |
|  | TAI | 7.827 | **0.006** | 0.035 |
|  | Health score | 1.042 | 0.308 | 0.005 |
|  | Priming * sex | 0.076 | 0.783 | 0.000 |
|  | Period * priming | 0.581 | 0.447 | 0.003 |
|  | Period * age | 3.101 | 0.080 | 0.014 |
|  | Period * TAI | 5.719 | **0.018** | 0.026 |
|  | Period * health score | 0.011 | 0.918 | 0.000 |
|  | Period * sex | 1.275 | 0.260 | 0.006 |
|  | Period * priming * sex | 0.822 | 0.365 | 0.004 |
| **BODS-external** | Period | 0.453 | 0.502 | 0.002 |
|  | Priming | 0.923 | 0.338 | 0.004 |
|  | Age | 1.498 | 0.222 | 0.007 |
|  | Sex | 4.699 | **0.031** | 0.021 |
|  | TAI | 2.732 | 0.100 | 0.012 |
|  | Health score | 0.707 | 0.401 | 0.003 |
|  | Priming * sex | 0.491 | 0.484 | 0.002 |
|  | Period * priming | 0.205 | 0.652 | 0.001 |
|  | Period * age | 1.846 | 0.176 | 0.008 |
|  | Period * TAI | 3.111 | 0.079 | 0.014 |
|  | Period * health score | 0.244 | 0.622 | 0.001 |
|  | Period * sex | 0.042 | 0.837 | 0.000 |
|  | Period * priming * sex | 0.011 | 0.915 | 0.000 |
| **C-DIS** | Period | 0.545 | 0.461 | 0.003 |
|  | Priming | 0.734 | 0.393 | 0.004 |
|  | Age | 5.247 | **0.023** | 0.026 |
|  | Sex | 6.319 | **0.013** | 0.031 |
|  | TAI | 5.027 | **0.026** | 0.025 |
|  | Health score | 2.391 | 0.124 | 0.012 |
|  | Priming * sex | 0.017 | 0.895 | 0.000 |
|  | Period * priming | 4.63e-4 | 0.983 | 0.000 |
|  | Period * age | 0.174 | 0.677 | 0.001 |
|  | Period * TAI | 0.189 | 0.664 | 0.001 |
|  | Period * health score | 0.943 | 0.333 | 0.005 |
|  | Period * sex | 1.499 | 0.222 | 0.007 |
|  | Period * priming * sex | 0.151 | 0.698 | 0.001 |

*Table S6a:* *Series of the repeated measure ANCOVAs testing the effect of the period (high or low pathogen threat), the priming condition, and sex on TDDS, BODS, and C-DIS scores controlling for age, health score and TAI. Df is 1 for all variables and 219 for residuals. Statistically significant associations are marked in bold.*

We also found a significant difference between female and male respondents in TAI, however, again there was no interaction between and period or/and priming, indicating that the difference is stable and not changing depending on the period of pathogen threat or used priming, see Table S6b.

| **Variable** | | **F** | **p** | **η^2^_p_** |
| --- | --- | --- | --- | --- |
| **TAI** | Period | 0.355 | 0.551 | 0.002 |
|  | Priming | 0.169 | 0.681 | 0.001 |
|  | Sex | 10.366 | **0.001** | 0.045 |
|  | Priming * sex | 4.10e-6 | 0.998 | 0.000 |
|  | Period * sex | 0.230 | 0.632 | 0.001 |
|  | Period * priming | 0.098 | 0.754 | 0.000 |
|  | Period * priming * sex | 0.208 | 0.649 | 0.001 |

*Table S6b: ANCOVA model testing the effect of the period (high or low pathogen threat), the priming condition and sex on TAI scores. Df is 1 for all variables and 220 for residuals. Statistically significant associations are marked in bold.*

**S7:** DIFFERENCES IN TRAIT VARIABLES BETWEEN THE PERIODS OF HIGH PATHOGEN THREAT AND LOW PATHOGEN THREAT

The ANCOVA with age as a covariate (F_1,445_ = 9.994, p = 0.002, η^2^_p_ = 0.022) showed no statistically significant differences in TAI scores between high and low pathogen threat periods (F_1,445_ = 0.088, p = 0.766, η^2^_p_ = 0.000). There was also no significant difference in health scores, F_1,445_ = 1.43, p = 0.232, η^2^_p_ = 0.022 (covariate age: F_1,445_ = 6.51, p = 0.011, η^2^_p_ = 0.022) between the periods of high and low pathogen threat.

**S8:** ASSOCIATIONS BETWEEN DISGUST MEASURES AND COVID CONCERNS AND AVOIDANCE

|  | **High pathogen threat** | | | | **Low pathogen threat** | | | |
| --- | --- | --- | --- | --- | --- | --- | --- | --- |
|  | **Covid concerns** | | **Covid avoidance** | | **Covid concerns** | | **Covid avoidance** | |
|  | **r** | **p** | **r** | **p** | **r** | **p** | **r** | **p** |
| **TDDS total score** | 0.222 | **<0.001** | -0.004 | 0.949 | 0.154 | **0.024** | 0.044 | 0.527 |
| **TDD-pathogen** | 0.193 | **0.004** | -0.016 | 0.811 | 0.067 | 0.328 | -0.061 | 0.381 |
| **TDD-moral** | 0.161 | **0.017** | -0.008 | 0.900 | 0.105 | 0.124 | 0.026 | 0.715 |
| **TDD-sexual** | 0.135 | **0.045** | -0.022 | 0.748 | 0.160 | **0.019** | 0.135 | 0.052 |
| **BODS-internal** | -0.005 | 0.937 | -0.155 | **0.021** | 0.046 | 0.501 | -0.005 | 0.939 |
| **BODS-external** | 0.161 | **0.017** | -0.031 | 0.644 | 0.081 | 0.239 | -0.063 | 0.370 |
| **C-DIS** | 0.232 | **<0.001** | 0.048 | 0.532 | 0.089 | 0.209 | -0.051 | 0.482 |

*Table S8:* *Partial Pearson’s correlation between disgust measures, covid concerns, and covid avoidance controlled for age.*
